# A Previously Unreported Variant of a Thoracic Vertebra

**DOI:** 10.7759/cureus.1522

**Published:** 2017-07-27

**Authors:** Vlad Voin, Michele Davis, Marcus A Cox, Marios Loukas, Jens Chapman, Rod J Oskouian, R. Shane Tubbs

**Affiliations:** 1 Anatomical Research, Seattle Science Foundation, Seattle, USA; 2 Department of Anatomical Sciences, St. George's University School of Medicine, St. George’s, GRD; 3 Internal Medicine, Saint Michael's Medical Center, Newark, USA; 4 Medical Education and Simulation, St. George's University School of Medicine, St. George, GRD; 5 Neurosurgery, Swedish Neuroscience Institute, Seattle, USA; 6 Departments of Neurosurgery and Structural & Cellular Biology, Tulane University & Ochsner Clinic Neurosurgery Program, Tulane University School of Medicine; St. George's University, Grenada, New Orleans, USA

**Keywords:** vertebrae, spine, transverse process, duplication, doubled

## Abstract

Pathology associated with anomalous transverse processes is uncommon and usually involves elongated C7 transverse processes in the so-called cervical rib syndrome. We report a single adult thoracic vertebra found to have duplicated transverse processes on the left side. We believe this to be the first report of a duplicated transverse process in man. The clinician and anatomist who view osteological material or imaging should consider this an extremely rare anatomical variation of the thoracic spine.

## Introduction

The transverse process is a bony protrusion that extends from the lateral edge of the neural arch and functions as a site of attachment of deep back muscles and ligaments. The cervical transverse processes are morphologically different from the rest of the spine due to the fact that they contain a foramen (foramen transversarium), which houses the vertebral artery [1]. Anomalies of the transverse process are rare occurrences, and there is a scarce amount of literature on this topic.

After an extensive literature search of PubMed, Embase, and Google Books, we identified a few papers that reported anomalies of the transverse process. Bergman, et al. state in their Illustrated Encyclopedia of Human Anatomic Variation, “the transverse process of the seventh vertebra may be bifid [2],” which is the only anatomic variant regarding such that is referenced. Another variation, documented by a few authors but still very rare, is elongation of the anterior part of the transverse process in the cervical spine [3-5]. According to the case reports, the transverse process anomaly presents as a bony projection anterior to the vertebral body found on imaging of the spine. In one case report, Shibayama, et al. associated a transverse process anomaly in the lumbar spine with Bertolotti’s syndrome [6].

Other than the few cases mentioned above, transverse process anomalies are very uncommon. Below is an example of an extremely rare case of a duplicate transverse process. To our knowledge, there has been no other documentation of a duplicate transverse process in English medical literature.

## Case presentation

**Figure 1:**
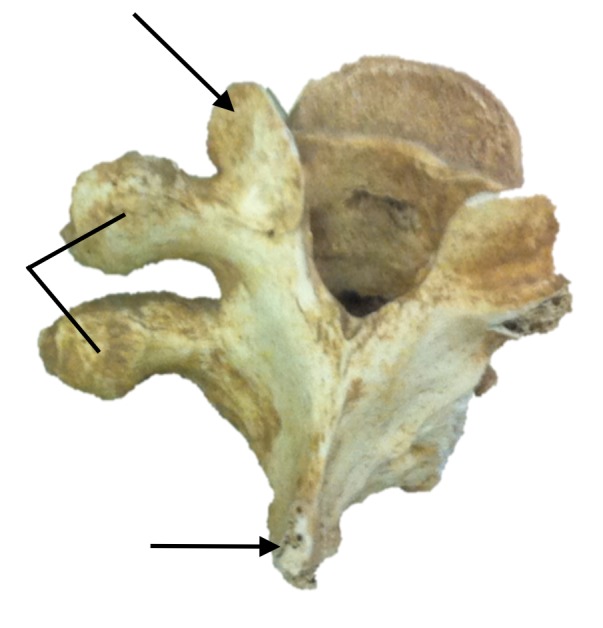
Specimen as described herein with duplication of the left transverse process (lines) of T10 vertebra. For reference, the left superior articular process (upper arrow) and spinous process (lower arrow) are indicated.

An extensive osteological collection from various universities identified a single specimen (sex unknown) of a thoracic vertebra, which was T10. The specimen was from an adult and had no unusual findings except that on the left side, the transverse process was duplicated (Figure 1). There were no findings of old fractures or other disease. Although no ribs were found with this isolated specimen, both transverse processes had articular facets present indicating that in life, ribs had articulated at each process. The left and right laminae of this vertebra were thought to be slightly elongated in the coronal plane from normal and the inferior articular facet was related to the lower transverse process and the superior articular facet was related to the base of the superior transverse process. The right side of this vertebra was thought to be normal although the “normal” transverse process was broken off. The vertebral canal and body were both found to be normal. Additionally, the left-sided intervertebral foramen of this level was not compromised although the two transverse processes were closer to each other than normal.

## Discussion

The development of the vertebral column begins during the fourth week of embryogenesis. Signals from the notochord induce and maintain proliferation of the sclerotome (part of the somite), and later promote chondrogenesis. The sclerotome surrounds the spinal cord and notochord forming a long mesenchymal column. The cells of the sclerotome then migrate in three different directions; ventromedially to form the vertebral body, dorsally to form the vertebral arch, and ventrolaterally to form the costal processes. Next, in week six, multiple centers of chondrification develop in the mesenchymal vertebrae and vertebral arches. The spinous and transverse processes develop from extensions of chondrification centers in the vertebral arch. And lastly, the ossification stage begins in the embryonic period and ends at about 25 years of age [7].

Mivart states that the development of the vertebral column is similar in humans as it is in other vertebrates, and the reason for variation of ribs and transverse processes in vertebrates is due to the arresting of the notochords at different stages. An example can be found in Lessons in Elementary Anatomy by St. George Jackson Mivart, which shows vertebrae having four ribs on each side originating from a doubled transverse process. The vertebrae came from a Polypterus, which is a genus of primitive freshwater fish found only in Africa and are characterized by numerous dorsal finlets [8].

Another etiology of what might have caused this duplicated transverse process is Klippel-Feil syndrome (KFS). This is a rare congenital skeletal malformation characterized by the triad of short neck, limitation of head and neck movements and low posterior hairline [9]. There have been three types of Klippel-Feil syndrome described with type three involving the thoracic and lumbar spine anomalies [10]. The etiology of KFS is still unknown, but there have been some associations with chromosomal translocations and teratogenic causes that could lead to abnormal formation of the vertebral column [9-10].

The fact that this specimen’s history is unknown to us makes it difficult to comment on the effect this duplicate transverse process had on the patient or if the patient had any other congenital abnormalities. Upon further review of the case reports concerning enlargement of the transverse process of the cervical vertebrae, which is hypothesized to be a form of supernumerary cervical ribs developing at a level above the lowest cervical vertebra, all the patients presented with different neurological complaints ranging from neck pain to numbness and tingling [3-5]. In all these case reports, there was no conclusive evidence to attribute the anomalies with the symptomatology of the patients. The findings of the enlarged transverse process were attributed as incidental findings on radiology [3-5]. However, in the case report involving the anomaly in the lumbar spine, they did find an association between the patient’s symptoms and an enlarged transverse process [6]. According to Shibayama, et al., Bertolotti’s syndrome is the enlargement of the transverse process of the fifth lumbar vertebra and is associated with lower back pain and sciatica [6]. He proposed that the signs and symptoms of Bertolotti’s syndrome were caused by impingement of the nerve root via the enlarged transverse process. This observation was made when microendoscopic decompression of the spinal canal did not resolve the patient’s pain and only when they microendoscopically decompressed the enlarged transverse process was the patient’s pain resolved [6].

## Conclusions

Although the anomaly is apparently very rare, if encountered in the future by surgeons or radiologists, having a reference to it in medical literature would be important. Additional reports are now necessary to better understand the true etiology and prevalence of such an osteological anomaly.
